# Supplementary material for: Content-rich biological network constructed by mining PubMed abstracts
Source: BMC Bioinformatics. 2004 Oct 8;5:147. doi: 10.1186/1471-2105-5-147 (PMC528731; doi:10.1186/1471-2105-5-147)
Supplement: Additional File 2 — The original results of the above study (non-essential files are deleted to keep the file size under the limit set by BMC bioinformatics). [file 1471-2105-5-147-S2.bz2 › chilibotAdditionalFile2/dip05/303ID9501253E1300/html/APP_A2M.html]

 


 **APP** and **A2M** 
  
Found 50 abstracts in PubMed, retrieved 05.  
 

 What does Google say? 
 PDF only 
| .edu only 

---

**Interactive relationship** (e.g. stimulation, inhibition, etc)

**Neutral relationship**- Haptoglobin Hp and alpha 2 macroglobulin  [ **A2M** ]  alpha 2 M  [ **A2M** ]  are among the most important  **APP**  in rats.  Ref: 11521461 Vojnosanit Pregl< MedlineTA>Vojnosanit Pregl,

**Non-interactive relationship** (e.g. studied together, co-existance, homology, etc.)

- Most of these studies have examined only one or two polymorphisms in a defined population, which provides a lack of a general view of the Alzheimer s disease genetic component.OBJECTIVETo overcome this limitation, nine polymorphisms in seven different candidate genes  **A2M** , ACT, APOE,  **APP** , BH, HSP70 2, and IL1 A were genotyped.METHODSThe sample comprised 112 Alzheimer s disease patients and 89 controls from Spain.  Ref: 12782964 Psychiatr Genet, 2003
